# Correction: Beyond wind speed: Integrating oceanic indices and time-lagged features for superior wind energy prediction

**DOI:** 10.1371/journal.pone.0347371

**Published:** 2026-04-14

**Authors:** Namal Rathnayake, Mahesh Yadav, Jeevani Jayasinghe, Upaka Rathnayake, Masashi Minamide, Yukinobu Hoshino

The second author’s name is spelled incorrectly. The correct name is: Mahesh Yadav. The correct citation is: Rathnayake N, Yadav M, Jayasinghe J, Rathnayake U, Minamide M, Hoshino Y (2026) Beyond wind speed: Integrating oceanic indices and time-lagged features for superior wind energy prediction. PLoS One 21(3): e0344167. https://doi.org/10.1371/journal.pone.0344167

There is an error in affiliation 4 for author Jeevani Jayasinghe. The correct affiliation 4 is: Department of Electronics, Faculty of Applied Sciences, Wayamba University of Sri Lanka, Kuliyapitiya, 60200, Sri Lanka.

The ORCID iD is missing for the third author. Please see the authors’ respective ORCID iD here:

Author Jeevani Jayasinghe’s ORCID iD is: 0000-0002-9266-8643 (https://orcid.org/0000-0002-9266-8643).

S1–S16 Tables are currently formatted as.tex files instead of PDF format. It can be viewed below.

## Supporting information

S1 Table Experiment A – Validation Results. This table presents the validation performance metrics for Experiment A.
(PDF)

S2 Table Experiment A – Test Results. This table presents the test performance metrics for Experiment A.
(PDF)

S3 Table Experiment A – Model Specifications. This table details the model configurations used in Experiment A.
(PDF)

S4 Table Experiment A – Hyperparams. This table lists the hyperparameter settings for Experiment A.
(PDF)

S5 Table Experiment B – Validation Results. This table presents the validation performance metrics for Experiment B.
(PDF)

S6 Table Experiment B – Test Results. This table presents the test performance metrics for Experiment B.
(PDF)

S7 Table Experiment B – Model Specifications. This table details the model configurations used in Experiment B.
(PDF)

S8 Table Experiment B – Hyperparams. This table lists the hyperparameter settings for Experiment B.
(PDF)

S9 Table Experiment C – Validation Results. This table presents the validation performance metrics for Experiment C.
(PDF)

S10 Table Experiment C – Test Results. This table presents the test performance metrics for Experiment C.
(PDF)

S11 Table Experiment C – Model Specifications. This table details the model configurations used in Experiment C.
(PDF)

S12 Table Experiment C – Hyperparams. This table lists the hyperparameter settings for Experiment C.
(PDF)

S13 Table Experiment D – Validation Results. This table presents the validation performance metrics for Experiment D.
(PDF)

S14 Table Experiment D – Test Results. This table presents the test performance metrics for Experiment D.
(PDF)

S15 Table Experiment D – Model Specifications. This table details the model configurations used in Experiment D.
(PDF)

S16 Table Experiment D – Hyperparams. This table lists the hyperparameter settings for Experiment D.
(PDF)
