# Supplementary material for: Beyond wind speed: Integrating oceanic indices and time-lagged features for superior wind energy prediction
Source: PLoS One. 2026 Mar 19;21(3):e0344167. doi: 10.1371/journal.pone.0344167 (PMC13001975; doi:10.1371/journal.pone.0344167)
Supplement: Attachment — Submitted filename: Response_to_Reviewer_3_R1.pdf [file pone.0344167.s021.pdf]

Response letter for manuscript Beyond Wind Speed: Integrating Oceanic Indices and Time-Lagged Features for Superior Wind Energy Prediction

July 21, 2025

Reviewer's Comments - 3

1 In the introduction, the problem statement and research gaps should be explained more in detail. The authors need to better explain the context of this research, including why the research problem is important. The introduction should clearly explain the key limitations of prior work that are relevant to this paper.

Authors' responses:

Thank you for your valuable suggestion. We have addressed this in the revised Introduction (pp. 2–3), where we now:

- Emphasize the operational challenges posed by wind's high temporal variability for grid stability and market efficiency.
- Highlight that most prior ML–based forecasting studies rely solely on local meteorological inputs and overlook broader climate drivers at seasonal to interannual scales.
- Note that existing work either considers individual teleconnection indices in isolation or neglects them, and that no previous study has integrated multiple indices within a unified ML framework for wind power prediction.

2 The manuscript needs a better literature review to delimit the research problem and leads the reader to the objectives of the study.

Authors' responses:

Thank you for your valuable feedback. We have thoroughly reorganized the Literature Review to more clearly delimit the research problem and lead the reader logically to our study objectives. The revised section now progresses from the general challenges of wind power forecasting to the specific gap in integrating multiple climate indices, and it features updated, high-quality references selected for their direct relevance to our topic.

3 Specific discussion is required like conclusion part. The discussion part seems like a curiosity. Here you should use the literature to support and discuss your results.

Authors' responses:

Thank you for your suggestion. In the revised Discussion, we now anchor our observations firmly in the literature, as shown below:

> The stepwise enhancement of predictive performance from Experiments A through D underscores the complementary roles of oceanic teleconnection indices, temporal depth, and principled feature selection. In Experiment B, the inclusion of contemporaneous indices modestly enriched the feature space but introduced significant noise, as many indices exhibit weak instantaneous correlation with monthly wind power; this aligns with prior findings that raw climate modes can degrade short-term forecasts unless their temporal structure is exploited [12,13]. Experiment C's full suite of 1- to 12-month lags harnesses the delayed coupling between ocean-atmosphere dynamics and regional wind patterns, compressing the RMSE spread by allowing models to learn autocorrelated signals, consistent with studies showing multi-month ENSO lags improve wind-speed forecasts [31]. Finally, Experiment D applies MRMR to distill 156 lagged predictors into the three most informative variables (`WIND`, `AMM_9`, `WIND_6`), eliminating redundant or spurious inputs; the resulting RMSE minima (approximately 50 MWh) and $R^2 \approx 0.99$ far exceed benchmarks in the literature and mirror the success of MRMR–PSO–LSTM in other energy-forecasting domains [10,17]. The demonstrable gains from integrating climate indices, lags, and MRMR selection yield immediate benefits for grid operators by reducing forecast uncertainty. More accurate monthly wind-power projections enable optimized dispatch scheduling, reduced reliance on expensive spinning reserves, and enhanced stability of mixed-renewable energy portfolios.

•

Note: The updated content is highlighted in the manuscript for your reference.
The authors appreciate all of the insightful comments from the Reviewer. We worked hard to be responsive to them. Thank you for taking the time and energy to help us improve the paper.

Thank you.
