# Supplementary material for: Beyond wind speed: Integrating oceanic indices and time-lagged features for superior wind energy prediction
Source: PLoS One. 2026 Mar 19;21(3):e0344167. doi: 10.1371/journal.pone.0344167 (PMC13001975; doi:10.1371/journal.pone.0344167)
Supplement: Attachment — Submitted filename: Review-PONE-D-25-17132R1.pdf [file pone.0344167.s019.pdf]

“Beyond Wind Speed: Integrating Oceanic Indices and Time-Lagged Features for Superior Wind Energy Prediction”

Paper: PONE-D-25-17132R1

This study presents a framework combining large-scale oceanic climate indices and time-lagged features with advanced machine learning models to improve short-term wind power predictions. The authors evaluated four configurations: (A) baseline with wind speed; (B) wind plus contemporaneous indices; (C) adding 1–12 month lags for wind and indices; and (D) MRMR-based feature selection. Results from 25 models on monthly data from the Pawan Danavi wind farm (2015–2019) showed that lagged features significantly reduce RMSE and improve R2. Key variables identified include current wind speed and lagged factors, achieving an RMSE of approximately 50 MWh and R2 of about 0.99. This approach offered efficient forecasts, supporting reliable grid operations and future renewable energy integration.

In summary, the topic of this article is both pertinent and important. The manuscript explores an intriguing subject relevant to the journal. It presents a new framework to address the challenge. The revised version shows significant improvements over the initial submission, with clearer methodology, better organization, and stronger results. However, the manuscript still requires further enhancements in the depth of discussion, inclusion of state-of-the-art comparisons, and improvements in language quality. Therefore, I suggest that the following points be considered before accepting it as a minor revision.

Problems:

1. Expand the Discussion section to include more analytical depth and connect findings to recent related studies.
2. Strengthen the evaluation by including comparisons with newer state-of-the-art methods. Why were certain recent models (e.g., transformer-based or hybrid deep learning approaches) not included in the comparative analysis?
3. The papers (https://doi.org/10.1038/s41598-024-70350-5), (https://doi.org/10.1007/s13369-023-07892-9) and (https://doi.org/10.1063/5.0164437) are related to the fields of wind turbines and machine learning. Study them and mention them in the introduction.
4. Improve the English writing and style for better fluency and clarity.

5. Add a concise section discussing the limitations and potential future work of the study.
6. Has the proposed method been tested on noisy or imbalanced datasets? If so, what were the results
7. How well does the model generalize to other datasets beyond the one currently studied?
