# Supplementary material for: Beyond wind speed: Integrating oceanic indices and time-lagged features for superior wind energy prediction
Source: PLoS One. 2026 Mar 19;21(3):e0344167. doi: 10.1371/journal.pone.0344167 (PMC13001975; doi:10.1371/journal.pone.0344167)
Supplement: Attachment — Submitted filename: PONE-D-25-17132.pdf [file pone.0344167.s020.pdf]

Summary and claimed contributions: PONE-D-25-17132

The paper underlines the question as to whether oceanic indices and lagged features on a larger scale could improve the wind-power forecast significantly for the months on the Sri Lankan single wind farm between 2015 and 2019. Four combinatory setups are reported at a time: A) wind itself; B) wind in conjunction with any climate indices for the month; C) wind in conjunction with 1–12-month lags for wind and indices; and D) being lagged together itself separately by the MRMR feature selection. The benchmark dataset, navigated across a broad 25-model landscape (linear, tree ensembles, SVMS, GPRS, feedforward NNs), helped us conclude that the best model (D) after MRMR contains three predictors: current wind speed, AMM at a 9-month lag, and wind speed at a 6-month lag, with RMSE ≈ 50 MWh and $R^2 \approx 0.99$, and maintained computational effectiveness. The study's main point is that raw indices by themselves appear to impair the forecast skill; it is the lags along with the MRMR that significantly enhances it.

The core idea and benchmarks are broad and strong, yet there needs to be clarification on forecast framing with respect to "forecasting" vs "contemporaneous modeling," the time-series validation protocol, and the physical interpretation of AMM as chosen teleconnection; additionally, part of the inference should be clarified or reconsidered to contain information leakage and prevent over-optimism in the skills.

The most critical information

(Forecast framing & target horizon)

1) The best feature set is made up of the wind speed of the current month, which leads to contemporaneous power modeling (aka power-curve mapping), and not forecasting per se. Please refine the definition for forecast horizon and de-select the wind speed of the same month from the predictors in case ahead-of-time prediction is intended, which also makes the horizon-specific results (e.g., h=1,3,6 months) relying only on information available at t−h. If not, present framing of nowcasting/power modeling explicitly.

Time-series validation & leakage control

2) With "folds (9:1)" proposed for training/validation., weekly data splits along an autocorrelation and seasonality-rich monthly time series may result in random k-fold CV beating the purpose for leakage. Please re-evaluate using time-ordered/blocked or rolling (walk-forward) cross-validation strategies and provide the performance dispersion across folds.

Sample size & overfitting risk

3) While MRMR further filters out predictors and models for a proper job of training and validation, the risk of overfitting still remains due to the long list of potential predictors. The aim is to show the robustness of validation, particularly under time block cross-validation, by specifying confidence or prediction intervals—modeling the confidence limits given GPR-type methods and model families. Additonal sections within the paper could include simpler climatology and persistence-based measurements to assess the raw skill.

4) On the Physical Rationale of the Teleconnections MRMR-represented AMM improving Khombo Hut is an interesting but not so transparent case. Show cross-correlation/ autocorrelation plots, partial correlations, and bring in an associated physical-routes analysis. Mention points of reference like Indo-Pacific indices (e.g., ENSO, IOD, MJO) to distinguish against spurious selection.

Baseline clarity & metric definitions

5) Give formal definitions to the main metrics when first mentioned (if not already in the main body).

6) The sqrt (Mean Squared Error) allows for a measure of the accuracy of fitting a particular model to actual data. Considering the impressive variance in window size

between models, a model can be judged for a specific accuracy filter. Grotesquely oversimplifying matters, RMSE will be presented.

$$\mathrm{RMSE} = \sqrt{\frac{1}{n}\sum_{i-1}^{n} (y_i - \hat{y}_i)^2}, R^2 = 1 - \frac{\sum_i (y_i - \hat{y}_i)^2}{\sum_i (y_i - \bar{y})^2}$$

7) MAE is another model evaluation measurement. Report against the mark, persistence, and climatology): and SkillMUP minus the skill against skill against Persistence/Climatology:

$$\mathrm{Skill} = 1 - \frac{\mathrm{RMSE_{model}}}{\mathrm{RMSE_{ref}}}$$

MRMR Description and Interpretations

8) An algorithmic description of the MRMR is given; the specification of the canonical difference criterion was suggested.

Consider adding the canonical difference criterion as the basic measure:

$$\max_S \frac{1}{|S|}\sum_{x \in S} I(x; y) - \frac{1}{|S|^2}\sum_{x_i, x_j \in S} I(x_i; x_j)$$

9) A model for MRMR can be complemented with post-hoc explainability to link the behavior of the retained features towards the prediction.

To align your work with an interpretable ML for coastal wind prediction, I suggest the two articles be molded into a contextual, complimentary citation:

10) Introduction / Related Work (after summarizing ML for wind, and climate-teleconnection inputs):

Port "Explainable deep learning techniques for wind speed forecasting in coastal areas: Integrating model configuration, regularization, early stopping, and SHAP analysis" (Neural Computing & Applications, 2025: “10.1007/s00521-025-11433-w”) for XAI practices (SHAP)

and the importance of model configuration/regularization/early stopping for feature selection when dealing with complex wind dynamics in coastal settings.

**Methods (Interpretability paragraph) or Discussion (Interpretation of selected features):

Fascilitating writing "Interpretable machine learning for coastal wind prediction: Integrating SHAP analysis and seasonal trends" (Journal of Coastal Conservation, 2025: “10.1007/s11852-025-01108-y”) this also implies that in this context a post-hoc SHAP technique would validate relevance for AMM-9 and WIND-6, illustrate the seasonal/diurnal effects (by analogy), and enhance operational relevance. The idea linked to performance is that the killing flaws of the raw index will permeate the performance, unless engineered properly, so interpretable ML will show when and why the climate signs help.

A sentence, though, cannot hurt from ringing out in the Discussion: "Our MRMR-select features could further be validated on SHAP-based attribution (see two references given) for practitioners to see how teleconnection phases and lagged winds shape monthly power, and to have better reconciliation of data-driven signals with physical reasoning."

Scope claims

11) It is proposed that the claim be responsibly indicated in the conclusion and disclaimed if accurate, addressing whether they were truly the “first” to incorporate climate modes into MRMR and wind-power forecasting.

Minor comments & presentation

For the given data frequency and the particular lead time, the word “short-term” generally includes sub-daily to weekly; for monthly data, one could talk about “sub-seasonal to seasonal.”

Model zoo: A list with 25 models is good; could you possibly squeeze a paragraph in the main text with a table on those models, their families, key hyper-parameters or working parameterization, cost for train and predict?

Uncertainty: When looking at GPRs, the evaluation will have to describe the interval coverage, like predictive intervals and validation metrics for integrated uncertainty, in order to certify the existence of a properly calibrated uncertainty.

Operations: The last paragraph on operational deployment is good, across which brief note should be added on a reserve reduction quantifiable through a simple-cost proxy (if feasible).
