# Supplementary material for: Beyond wind speed: Integrating oceanic indices and time-lagged features for superior wind energy prediction
Source: PLoS One. 2026 Mar 19;21(3):e0344167. doi: 10.1371/journal.pone.0344167 (PMC13001975; doi:10.1371/journal.pone.0344167)
Supplement: Attachment — Submitted filename: Wind_Plosone_R2_Reviwer_6.pdf [file pone.0344167.s022.pdf]

Response letter for manuscript Beyond Wind Speed: Integrating Oceanic Indices and Time-Lagged Features for Superior Wind Energy Prediction

November 30, 2025

Reviewer's Comments - 6

Thank you very much for acknowledging the strengths of our paper. We really appreciate them. Please see the following steps we took to address the weaknesses you mentioned. The modifications are colored in blue for your reference.

1 The best feature set includes the wind speed of the current month, which implies contemporaneous power modeling (nowcasting) rather than true ahead-of-time forecasting. Please refine the definition of the forecast horizon and remove same-month wind speed if ahead-of-time prediction is intended. Otherwise, explicitly frame the task as nowcasting/power modeling.

Authors' responses:

We thank the reviewer for this important clarification. Our study is designed as a *contemporaneous* ($h = 0$) monthly wind-power modeling task, where the target variable for month t is estimated using predictors available within the same month, including the current-month wind speed (WS_t). This setup corresponds to operational power-curve modeling or "nowcasting," and not to ahead-of-time forecasting. Therefore, the inclusion of WS_t is intentional and consistent with the problem formulation.

To avoid ambiguity, we have now clearly stated in the manuscript that: (1) the forecast horizon is $h = 0$; (2) we do not perform multi-step forecasting for $h = 1, 3, 6$; and (3) removing the contemporaneous wind-speed predictor would change the nature of the problem and is outside the scope of this work.

A new paragraph has been added to the manuscript (end of the "Climate indices and problem formulation" section) explicitly describing the nowcasting framework and distinguishing it from true ahead-of-time forecasting. This revision fully addresses the reviewer's concern.

2 With "folds (9:1)" proposed for training/validation, weekly data splits along an autocorrelation- and seasonality-rich monthly time series may result in random k-fold cross-validation defeating the purpose by introducing temporal leakage. Please re-evaluate using time-ordered/blocked or rolling (walk-forward) cross-validation strategies and provide the performance dispersion across folds.

Authors' responses:

We thank the reviewer for this important remark regarding temporal leakage. In the original version, the internal training/validation procedure was described using random 9:1 folds, which indeed can break the temporal structure of an autocorrelated monthly series. Following the reviewer's suggestion, we have revised the evaluation protocol as follows.

First, we have *replaced* the random 9:1 training/validation splits with a *time-ordered blocked cross-validation* scheme. The monthly observations are kept in chronological order, and each fold uses an earlier contiguous block of months for training and a subsequent, non-overlapping block for validation. No shuffling is applied, and future observations are never used to predict the past. This eliminates temporal leakage while still allowing multi-fold assessment of model robustness.

Second, we now report the *dispersion of performance across folds.* For each experimental setup (A–D) and the selected top-performing models, we compute the mean and standard deviation of RMSE and R^2 across the blocked folds. These summary statistics are reported in the revised manuscript (Section "Model Training and Evaluation") and in the new supplementary tables, allowing readers to quantify variability in predictive skill under the time-respecting cross-validation scheme.

Finally, we updated the methodological description and pseudocode for Experiments A–D to explicitly state that (i) training/validation splits are time-ordered/blocked, and (ii) the final independent test set remains strictly held out and chronologically subsequent to all cross-validation folds. We believe these revisions fully address the reviewer's concern about temporal leakage and provide a more rigorous evaluation of the proposed models.

3 While MRMR filters predictors and reduces model complexity, the risk of overfitting remains because of the large initial predictor space. The reviewer requests demonstration of validation robustness—particularly under time-block cross-validation—by providing confidence or prediction intervals (e.g., using GPR model families). Additional baseline comparisons such as climatology and persistence benchmarks are also suggested to contextualize raw skill.

Authors' responses:

We sincerely thank the reviewer for highlighting the importance of robustness assessment beyond point estimates. In the revised manuscript, we implemented several changes to address the concerns:

a) **Confidence and Prediction Intervals.** For all Gaussian Process Regression (GPR) models tested within Experiments A–D, we now report the 95% predictive intervals derived from the posterior variance of the GPR kernel. These intervals are presented both in tabular form and in a new figure (Section "Model Uncertainty and Predictive Intervals"), showing the mean prediction together with upper and lower confidence bounds across the time-block cross-validation folds. This addition provides transparent uncertainty quantification and directly addresses the risk of overfitting.

b) **Robustness Under Time-Blocked Cross-Validation.** Following the reviewer's suggestion, we now quantify robustness by reporting the dispersion (mean ± standard deviation) of RMSE and R^2 across the blocked CV folds. This allows direct evaluation of the model stability under temporally consistent training/validation splits.

c) **Climatology and Persistence Baselines.** We have added a new subsection, "Baseline Skill Metrics," in which we implement two simple but widely used reference models: (i) a *climatology* baseline equal to the long-term mean of the training period, and (ii) a *persistence* baseline where $\hat{WP}_t = WP_{t-1}$. These baselines are reported alongside the ML results, enabling readers to assess the raw skill of the proposed approach relative to simple, operationally meaningful heuristics.

d) **Discussion of Overfitting Risks.** We have expanded the Discussion section to explicitly address the residual risk of overfitting in the context of the initial high-dimensional predictor space. We clarify that although MRMR reduces redundancy, predictive intervals and fold-to-fold variability provide complementary evidence of model stability.

We believe these additions fully address the reviewer's concerns by improving transparency, demonstrating robustness, and grounding the improvements against simple, interpretable baseline models.

4 On the Physical Rationale of the Teleconnections MRMR-represented AMM improving Khombo Hut is an interesting but not so transparent case. Show cross-correlation/autocorrelation plots, partial correlations, and bring in an associated physical-routes analysis. Mention points of reference, such as Indo-Pacific indices (e.g., ENSO, IOD, MJO), to distinguish from spurious selection.

Authors' responses:

We thank the reviewer for this insightful comment and fully agree that the physical rationale behind the MRMR-selected Atlantic Meridional Mode (AMM) predictor should be made more transparent.

First, we have added a new subsection entitled *"Physical Interpretation of the AMM Teleconnection"* in the Results and Discussion. In this subsection, we present (i) cross-correlation and autocorrelation functions (ACF/CCF) for wind power, local wind speed, and AMM, and

(ii) partial correlations between wind power and AMM at multiple lags while controlling for major Indo–Pacific climate indices (e.g., ENSO, IOD, MJO). These analyses show that the nine-month AMM lag retains a statistically significant relationship with wind power even after conditioning on Indo–Pacific drivers, supporting the interpretation that its selection by MRMR is not spurious.

Second, we now include a brief *physical-routes* discussion, outlining how AMM-related north-tropical Atlantic SST anomalies can modulate large-scale circulation patterns (Hadley/Walker cells) and, in turn, influence the South Asian monsoon flow and low-level winds over the study region with a multi-month delay. This provides a qualitative dynamical explanation that is consistent with the observed lag structure highlighted by MRMR.

Third, to anchor the AMM pathway against better-known Indo–Pacific modes, we explicitly contrast the behavior of AMM with that of ENSO, IOD, and MJO. We show that, although Indo–Pacific indices do exhibit correlations with wind power, the combination of lag structure and partial-correlation analysis indicates that AMM contributes additional, non-redundant information about the slowly evolving large-scale background state. This explains why AMM_9 emerges as a key predictor alongside local wind speed and its lags.

We believe these additions clarify the physical plausibility of the AMM teleconnection and distinguish it from purely statistical artefacts, as requested by the reviewer.

5 Give formal definitions to the main metrics when first mentioned (if not already in the main body).

Authors' responses:

We thank the reviewer for this helpful suggestion. In the revised manuscript, we included a section called Performance Evaluation Metrics to introduce the evaluation metrics.

6 The sqrt (Mean Squared Error) allows for a measure of the accuracy of fitting a particular model to actual data. Considering the impressive variance in window size between models, a model can be judged for a specific accuracy filter. Grotesquely oversimplifying matters, RMSE will be presented.

Authors' responses:

We appreciate the reviewer's emphasis on the role of $\text{RMSE} = \sqrt{\text{MSE}}$ as an interpretable accuracy measure that enables direct comparison across models with differing functional complexity and receptive-field (window) sizes. In the revised manuscript, we clarify this rationale by explicitly stating why RMSE is used as the primary scalar accuracy criterion across all experimental configurations (A–D).

Furthermore, to avoid any oversimplification, we now (i) provide formal definitions of all evaluation metrics at their first appearance, (ii) report RMSE alongside R^2 and MAE to capture complementary aspects of model error structure, and (iii) include dispersion (mean ± standard deviation) of RMSE under time-block cross-validation to account for variability across

folds. These additions ensure that RMSE is used in an interpretable, statistically contextualized manner while still serving as the main comparative index across models, as suggested by the reviewer.

7 MAE is another model evaluation measurement. Report against the mark, persistence, and climatology): and SkillMUP minus the skill against skill against Persistence/Climatology:

Authors' responses:

We agree that MAE provides complementary insight and have added MAE values for all models and both baselines (persistence and climatology). However, we respectfully choose *not* to introduce the proposed SkillMUP metric for the following reasons.

First, SkillMUP represents a difference-of-skill-scores between two baselines, which is not a standard, widely interpretable measure in the wind-power forecasting literature. The difference between skills relative to climatology and persistence can be sensitive to small fluctuations in the MAE of either baseline, producing values that are difficult to interpret physically. Because our study emphasizes transparency, reproducibility, and comparability with prior work, we maintain the commonly accepted baseline skill formulation without derivations of higher-order differences.

Second, the two baselines already serve distinct and well-understood roles: climatology represents long-term mean behavior, whereas persistence represents short-memory autoregressive structure. Reporting MAE directly against both baselines already conveys the relative improvement clearly and avoids compounding uncertainties across baselines.

Third, to ensure rigorous evaluation without introducing non-standard indices, we instead focus on (i) MAE comparison with each baseline separately, and (ii) robustness under time-block cross-validation, which provides a more statistically grounded measure of generalization performance.

For these reasons, and to maintain methodological clarity, we report MAE and baseline comparisons but do not adopt the SkillMUP metric. We believe this approach fully addresses the reviewer's concerns while keeping the evaluation framework aligned with established practice in wind-power forecasting.

8 An algorithmic description of the MRMR is given; the specification of the canonical difference criterion was suggested. Consider adding the canonical difference criterion as the basic measure:

Authors' responses:

We appreciate the reviewer's suggestion regarding the canonical difference criterion for MRMR. However, we respectfully choose not to include this variant for the following reasons.

First, our implementation uses the classical "max-dependency" MRMR formulation based on mutual information, which is the most widely employed version in forecasting, hydrology, and climate-related machine-learning literature. Introducing an alternative criterion—such

as the canonical difference formulation—would expand the methodological space substantially and potentially distract from our main objective: evaluating the relative benefit of incorporating lagged teleconnection features under a consistent feature-selection framework.

Second, canonical-difference MRMR is mathematically equivalent to a reweighted form of the same mutual-information objective. Its inclusion would not change the selected features in our dataset (where redundancy is already handled through strong correlation filtering and time-lag construction) and would therefore not affect the empirical findings. For this reason, adding it would increase methodological complexity without substantive scientific benefit.

Third, to maintain clarity and reproducibility, we aim to limit the analysis to the standard MRMR formulation that is already well understood, computationally efficient, and aligned with prior studies in related forecasting domains. This preserves conceptual continuity and avoids introducing an additional hyperparameter or criterion that may overcomplicate the interpretation of results.

For these reasons, we retain the classical MRMR formulation and do not add the canonical difference criterion. We believe this keeps the methodology focused and consistent without affecting the scientific conclusions.

9 A model for MRMR can be complemented with post-hoc explainability to link the behavior of the retained features towards the prediction.

Authors' responses:

We agree that post-hoc explainability approaches (e.g., SHAP, LIME, partial dependence) can offer additional interpretability. However, we intentionally choose not to integrate these methods in the present study for the following reasons.

First, the central aim of this work is to evaluate the utility of incorporating lagged climate teleconnections under a consistent feature-engineering and selection framework. MRMR itself already provides explicit interpretability through mutual-information relevance and redundancy minimisation, which directly identifies the most informative predictors. Adding post-hoc explainability would introduce an additional modelling layer that is not essential for answering the core research question.

Second, post-hoc explainability methods, particularly SHAP, are known to be sensitive to correlation and multicollinearity structures. Because our predictor set involves lagged and temporally correlated climate indices, applying SHAP-like methods without substantial decorrelation preprocessing may lead to unstable or potentially misleading explanations. Ensuring methodological rigour in such an extension would require a dedicated study beyond the scope of this work.

Third, in the revised manuscript we already provide transparency by reporting (i) lag-specific correlation analysis, (ii) partial correlations controlling for major Indo–Pacific modes, and (iii) physical teleconnection rationale for the MRMR-selected AMM_9. These analyses sufficiently clarify why the selected features influence the predictive model without adding a secondary post-hoc layer.

For these reasons, and to maintain a focused and coherent scope, we do not incorporate additional post-hoc explainability techniques in this paper.

10 Introduction / Related Work (after summarizing ML for wind, and climate teleconnection inputs)

Authors' responses:

We thank the reviewer for these valuable references. In the revised manuscript, we have *added both suggested citations* to the Introduction, specifically within the related-work section that covers machine learning for wind forecasting and the role of teleconnection features. These works are now used to contextualize recent developments in explainable deep learning, highlight the relevance of model configuration and regularization strategies, and discuss how interpretable ML has been applied in coastal wind environments.

In addition, we have incorporated a brief discussion in the Methods/Discussion clarifying how these studies motivate the importance of well-engineered inputs and feature-selection pipelines when dealing with complex wind dynamics. While we deliberately do not introduce a new SHAP-based post-hoc analysis in this paper (as explained in Response 9), the cited works now serve as conceptual anchors that reinforce the rationale for using structured feature engineering (lags, MRMR, climate indices) to avoid the pitfalls associated with raw teleconnection inputs.

These additions address the reviewer's recommendation while maintaining the scope of our analysis consistent with the focus of the present study.

11 Minor comments & presentation For the given data frequency and the particular lead time, the word "short-term" generally includes sub-daily to weekly; for monthly data, one could talk about "sub-seasonal to seasonal." Model zoo: A list with 25 models is good; could you possibly squeeze a paragraph in the main text with a table on those models, their families, key hyper-parameters or working parameterization, cost for train and predict? Uncertainty: When looking at GPRs, the evaluation will have to describe the interval coverage, like predictive intervals and validation metrics for integrated uncertainty, in order to certify the existence of a properly calibrated uncertainty. Operations: The last paragraph on operational deployment is good, across which brief note should be added on a reserve reduction quantifiable through a simple-cost proxy (if feasible).

Authors' responses:

We thank the reviewer for these helpful presentation-related suggestions. All requested revisions have been implemented in the updated manuscript.

-

Note:
We sincerely thank the reviewer for their constructive recommendations, which helped strengthen the manuscript.

The authors appreciate the Reviewer's insightful comments. We worked hard to be responsive to them. Thank you for taking the time and energy to help us improve the paper.

Thank you.
