# Supplementary material for: Correction: Beyond wind speed: Integrating oceanic indices and time-lagged features for superior wind energy prediction
Source: PLoS One. 2026 Apr 14;21(4):e0347371. doi: 10.1371/journal.pone.0347371 (PMC13078619; doi:10.1371/journal.pone.0347371)
Supplement: S1 Table — This table presents the validation performance metrics for Experiment A. (PDF) [file pone.0347371.s001.pdf]

Supplementary file 1: Beyond Wind Speed: Integrating Oceanic Indices and Time-Lagged Features for Superior Wind Energy Prediction

Namal Rathnayake[1,*], Mahesh Yadev[2], Jeevani Jayasinghe[3], Upaka Rathnayake[4], Masashi Minamide[1], and Yukinobu Hoshino[5]

[1]Graduate School of Engineering, Faculty of Engineering, University of Tokyo, Hongo, Tokyo, 113-8656, Japan
[2]Ministry of Water Supply, Irrigation and Energy, Koshi Province, C7PG+924, Nepal
[3]Department of Electronics, Faculty of Engineering, Wayamba University, Kurunegala, 60170, Sri Lanka
[4]Department of Civil Engineering and Construction, Faculty of Engineering and Design, Atlantic Technological University, Sligo, F91 YW50, Ireland
[5]School of Systems Engineering, Kochi University of Technology, 185 Miyanokuchi, Tosayamada, Kami City, Kochi 782-8502, Japan

Contents

List of Tables

1 Experiment A - Validation Results 2

Sup.Table 1: Experiment A - Validation Results

Model Number	Model	RMSE	MSE	R2	MAE	MAPE %
1	Bagged Trees	277.18	76826.12	0.91	183.46	29.51
2	Bilayered Neural Network	261.74	68508.10	0.92	186.06	29.06
3	Boosted Trees	268.98	72350.05	0.91	181.36	26.43
4	Coarse Gaussian SVM	245.96	60497.17	0.93	178.59	31.93
5	Coarse Tree	908.18	824787.77	0.00	830.50	167.58
6	Cubic SVM	442.26	195589.55	0.76	343.39	51.10
7	Efficient Linear Least Squares	264.49	69954.20	0.92	204.82	44.50
8	Efficient Linear SVM	819.75	671990.14	0.19	649.33	84.97
9	Exponential GPR	254.70	64872.50	0.92	177.44	28.11
10	Fine Gaussian SVM	344.63	118767.80	0.86	220.61	37.49
11	Fine Tree	299.81	89888.12	0.89	208.36	29.54
12	Least Squares Regression Kernel	339.75	115429.68	0.86	239.42	43.52
13	Linear	264.38	69896.29	0.92	204.75	44.81
14	Linear SVM	641.97	412130.65	0.50	489.00	62.70
15	Matern 5/2 GPR	239.04	57141.20	0.93	164.85	28.01
16	Medium Gaussian SVM	258.19	66661.58	0.92	186.12	32.66
17	Medium Neural Network	311.23	96863.08	0.88	205.94	36.15
18	Medium Tree	377.73	142676.25	0.83	260.39	36.91
19	Narrow Neural Network	266.58	71066.19	0.91	192.78	33.50
20	Quadratic SVM	407.75	166259.97	0.80	327.18	58.87
21	Rational Quadratic GPR	240.26	57722.47	0.93	164.91	28.00
22	Squared Exponential GPR	240.25	57722.46	0.93	164.91	28.00
23	SVM Kernel	1012.89	1025949.21	-0.24	800.40	105.43
24	Trilayered Neural Network	228.56	52237.99	0.94	153.93	27.56
25	Wide Neural Network	305.30	93209.56	0.89	204.72	32.12
