# Supplementary material for: Correction: Beyond wind speed: Integrating oceanic indices and time-lagged features for superior wind energy prediction
Source: PLoS One. 2026 Apr 14;21(4):e0347371. doi: 10.1371/journal.pone.0347371 (PMC13078619; doi:10.1371/journal.pone.0347371)
Supplement: S2 Table — This table presents the test performance metrics for Experiment A. (PDF) [file pone.0347371.s002.pdf]

Supplementary file 2: Beyond Wind Speed: Integrating Oceanic Indices and Time-Lagged Features for Superior Wind Energy Prediction

Namal Rathnayake[1,*], Mahesh Yadev[2], Jeevani Jayasinghe[3], Upaka Rathnayake[4], Masashi Minamide[1], and Yukinobu Hoshino[5]

[1]Graduate School of Engineering, Faculty of Engineering, University of Tokyo, Hongo, Tokyo, 113-8656, Japan
[2]Ministry of Water Supply, Irrigation and Energy, Koshi Province, C7PG+924, Nepal
[3]Department of Electronics, Faculty of Engineering, Wayamba University, Kurunegala, 60170, Sri Lanka
[4]Department of Civil Engineering and Construction, Faculty of Engineering and Design, Atlantic Technological University, Sligo, F91 YW50, Ireland
[5]School of Systems Engineering, Kochi University of Technology, 185 Miyanokuchi, Tosayamada, Kami City, Kochi 782-8502, Japan

Contents

List of Tables

1 Experiment A - Test Results 2

Sup.Table 1: Experiment A - Test Results

Model Number	Model	MAE	MSE	RMSE	R2	MAPE %
1	Bagged Trees	210.02	137712.73	371.10	0.68	18.09
2	Bilayered Neural Network	188.60	113237.62	336.51	0.74	18.58
3	Boosted Trees	212.60	142020.90	376.86	0.67	18.96
4	Coarse Gaussian SVM	256.45	125381.24	354.09	0.71	26.57
5	Coarse Tree	626.48	435305.16	659.78	0.00	82.48
6	Cubic SVM	304.41	126708.64	355.96	0.71	28.42
7	Efficient Linear Least Squares	254.33	133877.94	365.89	0.69	27.72
8	Efficient Linear SVM	442.96	335204.11	578.97	0.23	36.76
9	Exponential GPR	186.62	95658.35	309.29	0.78	17.74
10	Fine Gaussian SVM	193.37	85631.96	292.63	0.80	19.51
11	Fine Tree	228.31	115613.14	340.02	0.73	18.18
12	Least Squares Regression Kernel	213.17	106502.99	326.35	0.75	22.77
13	Linear	254.00	134753.85	367.09	0.69	27.64
14	Linear SVM	494.05	418601.08	646.99	0.04	39.84
15	Matern 5/2 GPR	236.31	145502.62	381.45	0.66	22.99
16	Medium Gaussian SVM	227.47	125578.90	354.37	0.71	22.35
17	Medium Neural Network	245.03	142768.18	377.85	0.67	23.46
18	Medium Tree	179.36	75047.89	273.95	0.83	14.78
19	Narrow Neural Network	249.45	143870.48	379.30	0.67	24.53
20	Quadratic SVM	292.23	119365.56	345.49	0.72	27.03
21	Rational Quadratic GPR	241.83	149953.30	387.24	0.65	23.42
22	Squared Exponential GPR	241.83	149953.30	387.24	0.65	23.42
23	SVM Kernel	615.10	636156.31	797.59	-0.47	48.94
24	Trilayered Neural Network	211.92	133601.74	365.52	0.69	19.92
25	Wide Neural Network	246.56	143840.26	379.26	0.67	23.58
