# Supplementary material for: Correction: Beyond wind speed: Integrating oceanic indices and time-lagged features for superior wind energy prediction
Source: PLoS One. 2026 Apr 14;21(4):e0347371. doi: 10.1371/journal.pone.0347371 (PMC13078619; doi:10.1371/journal.pone.0347371)
Supplement: S3 Table — This table details the model configurations used in Experiment A. (PDF) [file pone.0347371.s003.pdf]

Supplementary file 3: Beyond Wind Speed: Integrating Oceanic Indices and Time-Lagged Features for Superior Wind Energy Prediction

Namal Rathnayake[1,*], Mahesh Yadev[2], Jeevani Jayasinghe[3], Upaka Rathnayake[4], Masashi Minamide[1], and Yukinobu Hoshino[5]

[1]Graduate School of Engineering, Faculty of Engineering, University of Tokyo, Hongo, Tokyo, 113-8656, Japan
[2]Ministry of Water Supply, Irrigation and Energy, Koshi Province, C7PG+924, Nepal
[3]Department of Electronics, Faculty of Engineering, Wayamba University, Kurunegala, 60170, Sri Lanka
[4]Department of Civil Engineering and Construction, Faculty of Engineering and Design, Atlantic Technological University, Sligo, F91 YW50, Ireland
[5]School of Systems Engineering, Kochi University of Technology, 185 Miyanokuchi, Tosayamada, Kami City, Kochi 782-8502, Japan

Contents

List of Tables

1 Experiment A - Model Specifications 2

Sup.Table 1: Experiment A - Model Specifications

Model Number	Model	Prediction Speed (obs/sec)	Training Time (sec)	Compact Model Size (bytes)	Coder Model Size (bytes)
1	Bagged Trees	1959.94	3.43	95474	17284
2	Bilayered Neural Network	10432.77	3.41	6644	2700
3	Boosted Trees	2195.58	1.58	97982	18266
4	Coarse Gaussian SVM	10181.57	3.44	3790	946
5	Coarse Tree	10654.46	1.05	2085	216
6	Cubic SVM	10000.56	3.01	3922	1074
7	Efficient Linear Least Squares	10827.94	0.89	9337	480
8	Efficient Linear SVM	10445.48	3.07	9369	480
9	Exponential GPR	10514.84	0.86	8533	3382
10	Fine Gaussian SVM	10768.34	0.83	3870	1026
11	Fine Tree	10569.58	1.06	3849	958
12	Least Squares Regression Kernel	7772.47	0.84	7341	1168
13	Linear	9434.29	3.21	3852	543
14	Linear SVM	8984.43	3.21	4178	1330
15	Matern 5/2 GPR	9962.36	3.31	8527	3376
16	Medium Gaussian SVM	10030.09	3.09	3790	946
17	Medium Neural Network	8731.51	2.96	5232	1812
18	Medium Tree	9181.8	3.76	2589	428
19	Narrow Neural Network	10842.07	1.52	4872	1452
20	Quadratic SVM	10494.81	0.87	3938	1090
21	Rational Quadratic GPR	7802.57	2.63	8578	3402
22	Squared Exponential GPR	6168.68	0.87	8547	3396
23	SVM Kernel	4137.58	3.13	7411	1168
24	Trilayered Neural Network	9728.15	1.15	8416	3948
25	Wide Neural Network	8175.87	1.79	7032	3612
