# Supplementary material for: Correction: Beyond wind speed: Integrating oceanic indices and time-lagged features for superior wind energy prediction
Source: PLoS One. 2026 Apr 14;21(4):e0347371. doi: 10.1371/journal.pone.0347371 (PMC13078619; doi:10.1371/journal.pone.0347371)
Supplement: S4 Table — This table lists the hyperparameter settings for Experiment A. (PDF) [file pone.0347371.s004.pdf]

Supplementary file 4:
Beyond Wind Speed: Integrating Oceanic Indices and Time-Lagged Features for Superior Wind Energy Prediction

Namal Rathnayake[1,*], Mahesh Yadev[2], Jeevani Jayasinghe[3], Upaka Rathnayake[4], Masashi Minamide[1], and Yukinobu Hoshino[5]

[1]Graduate School of Engineering, Faculty of Engineering, University of Tokyo, Hongo, Tokyo, 113-8656, Japan
[2]Ministry of Water Supply, Irrigation and Energy, Koshi Province, C7PG+924, Nepal
[3]Department of Electronics, Faculty of Engineering, Wayamba University, Kurunegala, 60170, Sri Lanka
[4]Department of Civil Engineering and Construction, Faculty of Engineering and Design, Atlantic Technological University, Sligo, F91 YW50, Ireland
[5]School of Systems Engineering, Kochi University of Technology, 185 Miyanokuchi, Tosayamada, Kami City, Kochi 782-8502, Japan

Contents

List of Tables

1 Experiment A - Hyperparams 2

Sup.Table 1: Experiment A - Hyperparams

Model Number	Model	Hyperparameters
1	Bagged Trees	Minimum leaf size: 8; Number of learners: 30; Number of predictors to sample: Select All
2	Bilayered Neural Network	Number of fully connected layers: 2; First layer size: 10; Second layer size: 10; Activation: ReLU; Iteration limit: 1000; Regularization strength (Lambda): 0; Standardize data: Yes
3	Boosted Trees	Minimum leaf size: 8; Number of learners: 30; Learning rate: 0.1; Number of predictors to sample: Select All
4	Coarse Gaussian SVM	Kernel function: Gaussian; Kernel scale: 4; Box constraint: Automatic; Epsilon: Auto; Standardize data: Yes
5	Coarse Tree	Minimum leaf size: 36; Surrogate decision splits: Off
6	Cubic SVM	Kernel function: Cubic; Kernel scale: Automatic; Box constraint: Automatic; Epsilon: Auto; Standardize data: Yes
7	Efficient Linear Least Squares	Learner: Least squares; Solver: Auto; Regularization: Auto; Regularization strength (Lambda): Auto; Relative coefficient tolerance (Beta tolerance): 0.0001;
8	Efficient Linear SVM	Learner: SVM; Solver: Auto; Regularization: Auto; Regularization strength (Lambda): Auto; Relative coefficient tolerance (Beta tolerance): 0.0001; Epsilon: Auto
9	Exponential GPR	Basis function: Constant; Kernel function: Exponential; Use isotropic kernel: Yes; Kernel scale: Automatic; Signal standard deviation: Automatic; Sigma: Automatic; Standardize data: Yes; Optimize numeric parameters: Yes
10	Fine Gaussian SVM	Kernel function: Gaussian; Kernel scale: 0.25; Box constraint: Automatic; Epsilon: Auto; Standardize data: Yes
11	Fine Tree	Minimum leaf size: 4; Surrogate decision splits: Off
12	Least Squares Regression Kernel	Learner: Least Squares Kernel; Number of expansion dimensions: Auto; Regularization strength (Lambda): Auto; Kernel scale: Auto; Standardize data: Yes; Iteration limit: 1000
13	Linear	Terms: Linear; Robust option: Off
14	Linear SVM	Kernel function: Linear; Kernel scale: Automatic; Box constraint: Automatic; Epsilon: Auto; Standardize data: Yes
15	Matern 5/2 GPR	Basis function: Constant; Kernel function: Matern 5/2; Use isotropic kernel: Yes; Kernel scale: Automatic; Signal standard deviation: Automatic; Sigma: Automatic; Standardize data: Yes; Optimize numeric parameters: Yes
16	Medium Gaussian SVM	Kernel function: Gaussian; Kernel scale: 1; Box constraint: Automatic; Epsilon: Auto; Standardize data: Yes
17	Medium Neural Network	Number of fully connected layers: 1; First layer size: 25; Activation: ReLU; Iteration limit: 1000; Regularization strength (Lambda): 0; Standardize data: Yes
18	Medium Tree	Minimum leaf size: 12; Surrogate decision splits: Off
19	Narrow Neural Network	Number of fully connected layers: 1; First layer size: 10; Activation: ReLU; Iteration limit: 1000; Regularization strength (Lambda): 0; Standardize data: Yes
20	Quadratic SVM	Kernel function: Quadratic; Kernel scale: Automatic; Box constraint: Automatic; Epsilon: Auto; Standardize data: Yes
21	Rational Quadratic GPR	Basis function: Constant; Kernel function: Rational Quadratic; Use isotropic kernel: Yes; Kernel scale: Automatic; Signal standard deviation: Automatic; Sigma: Automatic; Standardize data: Yes; Optimize numeric parameters: Yes
22	Squared Exponential GPR	Basis function: Constant; Kernel function: Squared Exponential; Use isotropic kernel: Yes; Kernel scale: Automatic; Signal standard deviation: Automatic; Sigma: Automatic; Standardize data: Yes; Optimize numeric parameters: Yes
23	SVM Kernel	Learner: SVM; Number of expansion dimensions: Auto; Regularization strength (Lambda): Auto; Kernel scale: Auto; Epsilon: Auto; Standardize data: Yes; Iteration limit: 1000
24	Trilayered Neural Network	Number of fully connected layers: 3; First layer size: 10; Second layer size: 10; Third layer size: 10; Activation: ReLU; Iteration limit: 1000; Regularization strength (Lambda): 0; Standardize data: Yes
25	Wide Neural Network	Number of fully connected layers: 1; First layer size: 100; Activation: ReLU; Iteration limit: 1000; Regularization strength (Lambda): 0; Standardize data: Yes
