# Supplementary material for: Correction: Beyond wind speed: Integrating oceanic indices and time-lagged features for superior wind energy prediction
Source: PLoS One. 2026 Apr 14;21(4):e0347371. doi: 10.1371/journal.pone.0347371 (PMC13078619; doi:10.1371/journal.pone.0347371)
Supplement: S5 Table — This table presents the validation performance metrics for Experiment B. (PDF) [file pone.0347371.s005.pdf]

Supplementary file 5: Beyond Wind Speed: Integrating Oceanic Indices and Time-Lagged Features for Superior Wind Energy Prediction

Namal Rathnayake[1,*], Mahesh Yadev[2], Jeevani Jayasinghe[3], Upaka Rathnayake[4], Masashi Minamide[1], and Yukinobu Hoshino[5]

[1]Graduate School of Engineering, Faculty of Engineering, University of Tokyo, Hongo, Tokyo, 113-8656, Japan
[2]Ministry of Water Supply, Irrigation and Energy, Koshi Province, C7PG+924, Nepal
[3]Department of Electronics, Faculty of Engineering, Wayamba University, Kurunegala, 60170, Sri Lanka
[4]Department of Civil Engineering and Construction, Faculty of Engineering and Design, Atlantic Technological University, Sligo, F91 YW50, Ireland
[5]School of Systems Engineering, Kochi University of Technology, 185 Miyanokuchi, Tosayamada, Kami City, Kochi 782-8502, Japan

Contents

List of Tables

1 Experiment B - Validation Results 2

Sup.Table 1: Experiment B - Validation Results

Model Number	Model	RMSE	MSE	R2	MAE	MAPE %
1	Bagged Trees	311.97	97324.99	0.88	221.74	39.52
2	Bilayered Neural Network	422.50	178503.58	0.78	323.59	48.96
3	Boosted Trees	300.30	90179.69	0.89	204.73	45.09
4	Coarse Gaussian SVM	610.52	372729.31	0.55	489.07	74.18
5	Coarse Tree	907.40	823370.65	0.00	830.05	38.95
6	Cubic SVM	999.51	999012.87	-0.21	806.86	82.59
7	Efficient Linear Least Squares	265.45	70463.40	0.91	202.69	41.73
8	Efficient Linear SVM	810.71	657255.13	0.20	639.68	41.39
9	Exponential GPR	344.01	118339.86	0.86	249.11	41.39
10	Fine Gaussian SVM	905.27	819513.71	0.00	821.59	54.56
11	Fine Tree	345.11	119101.78	0.86	238.57	35.41
12	Least Squares Regression Kernel	538.08	289528.64	0.65	439.08	33.19
13	Linear	281.81	79418.31	0.90	220.43	59.28
14	Linear SVM	1006.40	1012841.17	-0.23	813.94	90.20
15	Matern 5/2 GPR	262.98	69156.59	0.92	191.21	74.26
16	Medium Gaussian SVM	478.26	228728.70	0.72	351.56	65.71
17	Medium Neural Network	430.40	185242.77	0.78	333.60	82.06
18	Medium Tree	387.17	149902.94	0.82	267.33	61.11
19	Narrow Neural Network	452.42	204684.79	0.75	331.73	165.11
20	Quadratic SVM	1003.36	1006731.16	-0.22	810.81	167.85
21	Rational Quadratic GPR	260.47	67844.70	0.92	189.68	107.53
22	Squared Exponential GPR	260.47	67844.69	0.92	189.68	108.19
23	SVM Kernel	1007.21	1014472.97	-0.23	806.84	108.76
24	Trilayered Neural Network	522.98	273507.34	0.67	423.66	105.41
25	Wide Neural Network	397.04	157640.85	0.81	309.18	70.73
