# Supplementary material for: Correction: Beyond wind speed: Integrating oceanic indices and time-lagged features for superior wind energy prediction
Source: PLoS One. 2026 Apr 14;21(4):e0347371. doi: 10.1371/journal.pone.0347371 (PMC13078619; doi:10.1371/journal.pone.0347371)
Supplement: S6 Table — This table presents the test performance metrics for Experiment B. (PDF) [file pone.0347371.s006.pdf]

Supplementary file 6: Beyond Wind Speed: Integrating Oceanic Indices and Time-Lagged Features for Superior Wind Energy Prediction

Namal Rathnayake[1,*], Mahesh Yadev[2], Jeevani Jayasinghe[3], Upaka Rathnayake[4], Masashi Minamide[1], and Yukinobu Hoshino[5]

[1]Graduate School of Engineering, Faculty of Engineering, University of Tokyo, Hongo, Tokyo, 113-8656, Japan
[2]Ministry of Water Supply, Irrigation and Energy, Koshi Province, C7PG+924, Nepal
[3]Department of Electronics, Faculty of Engineering, Wayamba University, Kurunegala, 60170, Sri Lanka
[4]Department of Civil Engineering and Construction, Faculty of Engineering and Design, Atlantic Technological University, Sligo, F91 YW50, Ireland
[5]School of Systems Engineering, Kochi University of Technology, 185 Miyanokuchi, Tosayamada, Kami City, Kochi 782-8502, Japan

Contents

List of Tables

1 Experiment B - Test Results 2

Sup.Table 1: Experiment B - Test Results

Model Number	Model	MAE	MSE	RMSE	R2	MAPE %
1	Bagged Trees	248.83	151325.64	389.01	0.65	14.78
2	Bilayered Neural Network	388.40	261319.53	511.19	0.40	32.93
3	Boosted Trees	314.16	217488.75	466.36	0.50	40.78
4	Coarse Gaussian SVM	347.49	146908.42	383.29	0.66	41.42
5	Coarse Tree	626.48	435305.16	659.78	0.00	21.28
6	Cubic SVM	602.12	631932.48	794.94	-0.46	34.06
7	Efficient Linear Least Squares	296.46	137731.56	371.12	0.68	47.29
8	Efficient Linear SVM	443.64	339521.71	582.68	0.22	49.19
9	Exponential GPR	371.15	209089.73	457.26	0.52	49.19
10	Fine Gaussian SVM	590.47	378224.75	615.00	0.13	53.79
11	Fine Tree	296.11	231141.53	480.77	0.47	24.60
12	Least Squares Regression Kernel	460.00	262086.20	511.94	0.40	23.52
13	Linear	268.14	120068.50	346.51	0.72	48.29
14	Linear SVM	622.02	649419.70	805.87	-0.50	70.29
15	Matern 5/2 GPR	384.66	188539.16	434.21	0.57	63.48
16	Medium Gaussian SVM	417.39	286876.62	535.61	0.34	60.55
17	Medium Neural Network	531.43	356323.51	596.93	0.18	35.20
18	Medium Tree	179.36	75047.89	273.95	0.83	56.24
19	Narrow Neural Network	845.59	1029785.11	1014.78	-1.37	76.06
20	Quadratic SVM	617.13	644889.23	803.05	-0.49	82.48
21	Rational Quadratic GPR	399.68	199968.53	447.18	0.54	45.38
22	Squared Exponential GPR	399.68	199968.51	447.18	0.54	48.54
23	SVM Kernel	624.52	651128.51	806.93	-0.50	49.51
24	Trilayered Neural Network	352.67	164099.90	405.09	0.62	50.01
25	Wide Neural Network	491.75	305387.72	552.62	0.30	109.54
