# Supplementary material for: Correction: Beyond wind speed: Integrating oceanic indices and time-lagged features for superior wind energy prediction
Source: PLoS One. 2026 Apr 14;21(4):e0347371. doi: 10.1371/journal.pone.0347371 (PMC13078619; doi:10.1371/journal.pone.0347371)
Supplement: S7 Table — This table details the model configurations used in Experiment B. (PDF) [file pone.0347371.s007.pdf]

Supplementary file 7:
Beyond Wind Speed: Integrating Oceanic Indices and Time-Lagged Features for Superior Wind Energy Prediction

Namal Rathnayake[1,*], Mahesh Yadev[2], Jeevani Jayasinghe[3], Upaka Rathnayake[4], Masashi Minamide[1], and Yukinobu Hoshino[5]

[1]Graduate School of Engineering, Faculty of Engineering, University of Tokyo, Hongo, Tokyo, 113-8656, Japan
[2]Ministry of Water Supply, Irrigation and Energy, Koshi Province, C7PG+924, Nepal
[3]Department of Electronics, Faculty of Engineering, Wayamba University, Kurunegala, 60170, Sri Lanka
[4]Department of Civil Engineering and Construction, Faculty of Engineering and Design, Atlantic Technological University, Sligo, F91 YW50, Ireland
[5]School of Systems Engineering, Kochi University of Technology, 185 Miyanokuchi, Tosayamada, Kami City, Kochi 782-8502, Japan

Contents

List of Tables

1 Experiment B - Model Specifications 2

Sup.Table 1: Experiment B - Model Specifications

Model Number	Model	Prediction Speed (obs/sec)	Training Time (sec)	Compact Model Size (bytes)	Coder Model Size (bytes)
1	Bagged Trees	7583.098117	2.2705802	4076	1827
2	Bilayered Neural Network	6586.329707	3.181892	15520	6166
3	Boosted Trees	6076.154469	2.839795	11000	2055
4	Coarse Gaussian SVM	7290.105707	2.596463	9717	6873
5	Coarse Tree	1887.986462	1.9914953	143395	19733
6	Cubic SVM	8239.871824	2.4268193	10959	6403
7	Efficient Linear Least Squares	7152.412615	0.9443307	14942	9791
8	Efficient Linear SVM	6842.80555	4.049759	14962	9811
9	Exponential GPR	5015.604102	1.7993508	14993	9817
10	Fine Gaussian SVM	5556.870453	3.2504915	14948	9797
11	Fine Tree	2427.653673	3.7122355	144991	20355
12	Least Squares Regression Kernel	7878.381139	0.9367692	5336	2357
13	Linear	8244.904191	1.0948428	9187	5155
14	Linear SVM	6168.256325	2.7701933	12844	12671
15	Matern 5/2 GPR	7333.070791	1.642679	9093	6249
16	Medium Gaussian SVM	7692.965211	2.6832262	17495	13987
17	Medium Neural Network	8201.576525	0.9048303	11040	2055
18	Medium Tree	7830.172264	1.7875649	9095	5587
19	Narrow Neural Network	7204.610951	2.9084426	10237	7393
20	Quadratic SVM	7449.919982	3.2807197	3572	1615
21	Rational Quadratic GPR	6279.215796	1.8815449	10241	7393
22	Squared Exponential GPR	5321.874877	3.6040417	10241	7393
23	SVM Kernel	4913.6472	0.9324042	10329	7393
24	Trilayered Neural Network	6423.29515	1.7074747	12914	12671
25	Wide Neural Network	8089.039352	3.3284198	7415	3907
