# Supplementary material for: Correction: Beyond wind speed: Integrating oceanic indices and time-lagged features for superior wind energy prediction
Source: PLoS One. 2026 Apr 14;21(4):e0347371. doi: 10.1371/journal.pone.0347371 (PMC13078619; doi:10.1371/journal.pone.0347371)
Supplement: S9 Table — This table presents the validation performance metrics for Experiment C. (PDF) [file pone.0347371.s009.pdf]

Supplementary file 9:
Beyond Wind Speed: Integrating Oceanic Indices and Time-Lagged Features for Superior Wind Energy Prediction

Namal Rathnayake[1,*], Mahesh Yadev[2], Jeevani Jayasinghe[3], Upaka Rathnayake[4], Masashi Minamide[1], and Yukinobu Hoshino[5]

[1]Graduate School of Engineering, Faculty of Engineering, University of Tokyo, Hongo, Tokyo, 113-8656, Japan
[2]Ministry of Water Supply, Irrigation and Energy, Koshi Province, C7PG+924, Nepal
[3]Department of Electronics, Faculty of Engineering, Wayamba University, Kurunegala, 60170, Sri Lanka
[4]Department of Civil Engineering and Construction, Faculty of Engineering and Design, Atlantic Technological University, Sligo, F91 YW50, Ireland
[5]School of Systems Engineering, Kochi University of Technology, 185 Miyanokuchi, Tosayamada, Kami City, Kochi 782-8502, Japan

Contents

List of Tables

1 Experiment C - Validation Results 2

Sup.Table 1: Experiment C - Validation Results

Model Number	Model	RMSE	MSE	R2	MAE	MAPE %
1	Bagged Trees	350.50	122850.80	0.85	258.16	45.65
2	Bilayered Neural Network	500.64	250642.46	0.69	405.36	86.95
3	Boosted Trees	335.73	112712.62	0.86	225.50	45.69
4	Coarse Gaussian SVM	858.20	736506.18	0.07	711.03	124.83
5	Coarse Tree	892.04	795730.15	0.00	806.44	170.59
6	Cubic SVM	971.46	943729.66	-0.19	771.47	109.76
7	Efficient Linear Least Squares	687.76	473011.92	0.41	586.80	143.87
8	Efficient Linear SVM	485.26	235480.31	0.70	395.17	86.38
9	Exponential GPR	586.94	344499.61	0.57	479.94	113.56
10	Fine Gaussian SVM	891.38	794559.67	0.00	803.37	167.85
11	Fine Tree	404.69	163773.80	0.79	324.18	60.69
12	Least Squares Regression Kernel	737.18	543431.93	0.32	643.26	145.98
13	Linear	485.62	235826.49	0.70	359.67	82.91
14	Linear SVM	981.77	963878.00	-0.21	775.03	107.69
15	Matern 5/2 GPR	466.90	217997.28	0.73	368.02	86.53
16	Medium Gaussian SVM	631.26	398484.92	0.50	518.27	123.32
17	Medium Neural Network	476.26	226824.87	0.71	380.78	84.51
18	Medium Tree	364.25	132679.64	0.83	274.70	44.08
19	Narrow Neural Network	516.25	266514.20	0.67	405.86	89.42
20	Quadratic SVM	979.39	959204.74	-0.21	774.11	108.18
21	Rational Quadratic GPR	469.56	220489.28	0.72	371.59	87.47
22	Squared Exponential GPR	892.04	795730.15	0.00	806.44	170.59
23	SVM Kernel	985.10	970426.20	-0.22	776.24	106.64
24	Trilayered Neural Network	469.37	220305.01	0.72	392.05	78.78
25	Wide Neural Network	482.28	232592.93	0.71	384.90	82.45
