# Supplementary material for: Correction: Beyond wind speed: Integrating oceanic indices and time-lagged features for superior wind energy prediction
Source: PLoS One. 2026 Apr 14;21(4):e0347371. doi: 10.1371/journal.pone.0347371 (PMC13078619; doi:10.1371/journal.pone.0347371)
Supplement: S10 Table — This table presents the test performance metrics for Experiment C. (PDF) [file pone.0347371.s010.pdf]

Supplementary file 10:
Beyond Wind Speed: Integrating Oceanic Indices and Time-Lagged Features for Superior Wind Energy Prediction

Namal Rathnayake[1,*], Mahesh Yadev[2], Jeevani Jayasinghe[3], Upaka Rathnayake[4], Masashi Minamide[1], and Yukinobu Hoshino[5]

[1]Graduate School of Engineering, Faculty of Engineering, University of Tokyo, Hongo, Tokyo, 113-8656, Japan
[2]Ministry of Water Supply, Irrigation and Energy, Koshi Province, C7PG+924, Nepal
[3]Department of Electronics, Faculty of Engineering, Wayamba University, Kurunegala, 60170, Sri Lanka
[4]Department of Civil Engineering and Construction, Faculty of Engineering and Design, Atlantic Technological University, Sligo, F91 YW50, Ireland
[5]School of Systems Engineering, Kochi University of Technology, 185 Miyanokuchi, Tosayamada, Kami City, Kochi 782-8502, Japan

Contents

List of Tables

1 Experiment C - Test Results 2

Sup.Table 1: Experiment C - Test Results

Model Number	Model	MAE	MSE	RMSE	R2	MAPE %
1	Bagged Trees	132.66	38988.43	197.45	0.91	11.92
2	Bilayered Neural Network	336.49	135501.4	368.11	0.68	36.26
3	Boosted Trees	99.1	10176.53	100.88	0.98	11.52
4	Coarse Gaussian SVM	455.32	364140.08	603.44	0.13	29.13
5	Coarse Tree	581.46	436684.53	660.82	-0.04	57.37
6	Cubic SVM	621.16	642484.6	801.55	-0.54	42.21
7	Efficient Linear Least Squares	351.98	182915.92	427.69	0.56	29.51
8	Efficient Linear SVM	256.08	107996.94	328.63	0.74	17.8
9	Exponential GPR	207.88	82630.02	287.45	0.8	13.19
10	Fine Gaussian SVM	577.85	441456.94	664.42	-0.06	56.13
11	Fine Tree	306.44	125306.29	353.99	0.7	25.99
12	Least Squares Regression Kernel	353.3	181439.59	425.96	0.57	29.54
13	Linear	300.18	157786.88	397.22	0.62	36.5
14	Linear SVM	640.29	690137.14	830.74	-0.65	42.87
15	Matern 5/2 GPR	324.13	148372.29	385.19	0.65	38.37
16	Medium Gaussian SVM	267.91	111836.31	334.42	0.73	19.79
17	Medium Neural Network	343.67	130346.84	361.04	0.69	34.97
18	Medium Tree	222.55	198461.5	445.49	0.53	20.54
19	Narrow Neural Network	405.85	198822.32	445.89	0.52	39.51
20	Quadratic SVM	634.15	673694.52	820.79	-0.61	42.72
21	Rational Quadratic GPR	334.44	153530.85	391.83	0.63	39.38
22	Squared Exponential GPR	581.46	436684.53	660.82	-0.04	57.37
23	SVM Kernel	640.83	690704.39	831.09	-0.65	42.97
24	Trilayered Neural Network	360.26	154696.34	393.31	0.63	37.06
25	Wide Neural Network	407.13	202583.65	450.09	0.52	41.42
