# Supplementary material for: Correction: Beyond wind speed: Integrating oceanic indices and time-lagged features for superior wind energy prediction
Source: PLoS One. 2026 Apr 14;21(4):e0347371. doi: 10.1371/journal.pone.0347371 (PMC13078619; doi:10.1371/journal.pone.0347371)
Supplement: S11 Table — This table details the model configurations used in Experiment C. (PDF) [file pone.0347371.s011.pdf]

Supplementary file 11:
Beyond Wind Speed: Integrating Oceanic Indices and Time-Lagged Features for Superior Wind Energy Prediction

Namal Rathnayake[1,*], Mahesh Yadev[2], Jeevani Jayasinghe[3], Upaka Rathnayake[4], Masashi Minamide[1], and Yukinobu Hoshino[5]

[1]Graduate School of Engineering, Faculty of Engineering, University of Tokyo, Hongo, Tokyo, 113-8656, Japan
[2]Ministry of Water Supply, Irrigation and Energy, Koshi Province, C7PG+924, Nepal
[3]Department of Electronics, Faculty of Engineering, Wayamba University, Kurunegala, 60170, Sri Lanka
[4]Department of Civil Engineering and Construction, Faculty of Engineering and Design, Atlantic Technological University, Sligo, F91 YW50, Ireland
[5]School of Systems Engineering, Kochi University of Technology, 185 Miyanokuchi, Tosayamada, Kami City, Kochi 782-8502, Japan

Contents

List of Tables

1 Experiment C - Model Specifications 2

Sup.Table 1: Experiment C - Model Specifications

Model Number	Model	Prediction Speed (obs/sec)	Training Time (sec)	Compact Model Size (bytes)	Coder Model Size (bytes)
1	Bagged Trees	919.0547842	2.1801786	761911	40787
2	Bilayered Neural Network	1754.922967	2.3191711	43147	37963
3	Boosted Trees	933.0220367	3.2054466	761683	40689
4	Coarse Gaussian SVM	1511.460739	2.3098189	75205	72361
5	Coarse Tree	1526.950679	1.7418541	23708	20599
6	Cubic SVM	2427.102266	1.7944496	77721	74873
7	Efficient Linear Least Squares	1533.736861	2.2213512	34003	23343
8	Efficient Linear SVM	2093.578071	1.548973	34209	23343
9	Exponential GPR	1451.633594	2.4708306	85769	80629
10	Fine Gaussian SVM	1514.09517	2.4110752	80229	77385
11	Fine Tree	2533.062355	1.8855657	25220	21235
12	Least Squares Regression Kernel	1180.38151	2.5893868	96724	196645
13	Linear	1354.851313	3.6347679	706624	NaN
14	Linear SVM	1555.468739	2.459725	78961	74873
15	Matern 5/2 GPR	1864.919136	1.7848793	85763	80623
16	Medium Gaussian SVM	1435.649515	1.551896	72693	69849
17	Medium Neural Network	1599.535763	3.1054997	60335	55675
18	Medium Tree	1795.609526	2.4015117	24212	20811
19	Narrow Neural Network	2164.04465	1.7075303	41375	36715
20	Quadratic SVM	1654.017917	1.9138619	78977	76129
21	Rational Quadratic GPR	1370.055057	2.3956686	85814	80649
22	Squared Exponential GPR	1395.696063	3.3372784	85783	80643
23	SVM Kernel	1347.067153	1.6530232	96794	196645
24	Trilayered Neural Network	1878.106519	2.3345327	44919	39211
25	Wide Neural Network	1610.336111	1.7116661	155135	150475
