# Supplementary material for: Correction: Beyond wind speed: Integrating oceanic indices and time-lagged features for superior wind energy prediction
Source: PLoS One. 2026 Apr 14;21(4):e0347371. doi: 10.1371/journal.pone.0347371 (PMC13078619; doi:10.1371/journal.pone.0347371)
Supplement: S13 Table — This table presents the validation performance metrics for Experiment D. (PDF) [file pone.0347371.s013.pdf]

Supplementary file 13:
Beyond Wind Speed: Integrating Oceanic Indices and Time-Lagged Features for Superior Wind Energy Prediction

Namal Rathnayake[1,*], Mahesh Yadev[2], Jeevani Jayasinghe[3], Upaka Rathnayake[4], Masashi Minamide[1], and Yukinobu Hoshino[5]

[1]Graduate School of Engineering, Faculty of Engineering, University of Tokyo, Hongo, Tokyo, 113-8656, Japan
[2]Ministry of Water Supply, Irrigation and Energy, Koshi Province, C7PG+924, Nepal
[3]Department of Electronics, Faculty of Engineering, Wayamba University, Kurunegala, 60170, Sri Lanka
[4]Department of Civil Engineering and Construction, Faculty of Engineering and Design, Atlantic Technological University, Sligo, F91 YW50, Ireland
[5]School of Systems Engineering, Kochi University of Technology, 185 Miyanokuchi, Tosayamada, Kami City, Kochi 782-8502, Japan

Contents

List of Tables

1 Experiment D - Validation Results 2

Sup.Table 1: Experiment D - Validation Results

Model Number	Model	RMSE	MSE	R2	MAE	MAPE %
1	Bagged Trees	312.31	97535.99	0.88	207.50	36.42
2	Bilayered Neural Network	229.94	52872.09	0.93	174.28	30.16
3	Boosted Trees	307.05	94281.59	0.88	220.43	41.75
4	Coarse Gaussian SVM	262.44	68872.54	0.91	208.67	168.92
5	Coarse Tree	886.36	785641.55	0.00	803.06	106.31
6	Cubic SVM	921.38	848944.04	-0.08	732.20	104.88
7	Efficient Linear Least Squares	225.62	50905.24	0.94	174.32	103.04
8	Efficient Linear SVM	685.90	470462.62	0.40	540.85	115.96
9	Exponential GPR	192.03	36874.74	0.95	136.78	41.51
10	Fine Gaussian SVM	631.76	399115.15	0.49	512.73	41.29
11	Fine Tree	297.26	88362.47	0.89	220.59	36.19
12	Least Squares Regression Kernel	297.09	88260.80	0.89	221.69	84.51
13	Linear	225.54	50867.56	0.94	174.16	38.13
14	Linear SVM	966.01	933167.53	-0.19	765.39	32.18
15	Matern 5/2 GPR	179.01	32044.01	0.96	127.20	26.96
16	Medium Gaussian SVM	237.34	56331.43	0.93	178.16	28.33
17	Medium Neural Network	358.05	128200.87	0.84	231.53	30.05
18	Medium Tree	366.71	134474.41	0.83	263.37	26.96
19	Narrow Neural Network	282.69	79911.46	0.90	205.14	32.37
20	Quadratic SVM	951.42	905198.70	-0.15	753.09	70.38
21	Rational Quadratic GPR	172.79	29856.58	0.96	125.48	40.19
22	Squared Exponential GPR	172.79	29856.58	0.96	125.48	34.85
23	SVM Kernel	972.82	946383.33	-0.20	770.76	37.29
24	Trilayered Neural Network	235.30	55366.99	0.93	174.90	106.43
25	Wide Neural Network	234.30	54897.61	0.93	178.73	50.67
