# Supplementary material for: Correction: Beyond wind speed: Integrating oceanic indices and time-lagged features for superior wind energy prediction
Source: PLoS One. 2026 Apr 14;21(4):e0347371. doi: 10.1371/journal.pone.0347371 (PMC13078619; doi:10.1371/journal.pone.0347371)
Supplement: S14 Table — This table presents the test performance metrics for Experiment D. (PDF) [file pone.0347371.s014.pdf]

Supplementary file 14: Beyond Wind Speed: Integrating Oceanic Indices and Time-Lagged Features for Superior Wind Energy Prediction

Namal Rathnayake[1,*], Mahesh Yadev[2], Jeevani Jayasinghe[3], Upaka Rathnayake[4], Masashi Minamide[1], and Yukinobu Hoshino[5]

[1]Graduate School of Engineering, Faculty of Engineering, University of Tokyo, Hongo, Tokyo, 113-8656, Japan
[2]Ministry of Water Supply, Irrigation and Energy, Koshi Province, C7PG+924, Nepal
[3]Department of Electronics, Faculty of Engineering, Wayamba University, Kurunegala, 60170, Sri Lanka
[4]Department of Civil Engineering and Construction, Faculty of Engineering and Design, Atlantic Technological University, Sligo, F91 YW50, Ireland
[5]School of Systems Engineering, Kochi University of Technology, 185 Miyanokuchi, Tosayamada, Kami City, Kochi 782-8502, Japan

Contents

List of Tables

1 Experiment D - Test Results 2

Sup.Table 1: Experiment D - Test Results

Model Number	Model	MAE	MSE	RMSE	R2	MAPE %
1	Bagged Trees	148.13	66477.47	257.83	0.84	13.69
2	Bilayered Neural Network	130.19	25538.52	159.81	0.94	22.58
3	Boosted Trees	39.16	2878.84	53.65	0.99	20.54
4	Coarse Gaussian SVM	135.28	29170.56	170.79	0.93	57.37
5	Coarse Tree	581.46	436684.53	660.82	-0.04	41.66
6	Cubic SVM	560.03	529680.05	727.79	-0.27	40.32
7	Efficient Linear Least Squares	121.63	33074.46	181.86	0.92	38.23
8	Efficient Linear SVM	361.10	261583.98	511.45	0.37	26.26
9	Exponential GPR	131.26	27073.45	164.54	0.94	12.76
10	Fine Gaussian SVM	241.62	81561.27	285.59	0.81	13.22
11	Fine Tree	215.62	69346.61	263.34	0.83	13.68
12	Least Squares Regression Kernel	138.08	29582.14	171.99	0.93	22.50
13	Linear	120.94	33161.21	182.10	0.92	4.40
14	Linear SVM	623.25	657761.71	811.03	-0.57	13.14
15	Matern 5/2 GPR	136.17	24930.31	157.89	0.94	14.74
16	Medium Gaussian SVM	136.55	25747.86	160.46	0.94	14.77
17	Medium Neural Network	133.06	25779.60	160.56	0.94	12.42
18	Medium Tree	222.55	198461.50	445.49	0.53	14.74
19	Narrow Neural Network	177.66	56492.87	237.68	0.86	17.75
20	Quadratic SVM	601.99	615740.72	784.69	-0.47	15.01
21	Rational Quadratic GPR	133.57	24301.26	155.89	0.94	14.80
22	Squared Exponential GPR	133.57	24301.25	155.89	0.94	14.38
23	SVM Kernel	629.08	672849.81	820.27	-0.61	22.20
24	Trilayered Neural Network	213.32	85586.82	292.55	0.80	41.83
25	Wide Neural Network	132.40	25696.08	160.30	0.94	17.05
