# Supplementary material for: Correction: Beyond wind speed: Integrating oceanic indices and time-lagged features for superior wind energy prediction
Source: PLoS One. 2026 Apr 14;21(4):e0347371. doi: 10.1371/journal.pone.0347371 (PMC13078619; doi:10.1371/journal.pone.0347371)
Supplement: S15 Table — This table details the model configurations used in Experiment D. (PDF) [file pone.0347371.s015.pdf]

Supplementary file 15: Beyond Wind Speed: Integrating Oceanic Indices and Time-Lagged Features for Superior Wind Energy Prediction

Namal Rathnayake[1,*], Mahesh Yadev[2], Jeevani Jayasinghe[3], Upaka Rathnayake[4], Masashi Minamide[1], and Yukinobu Hoshino[5]

[1]Graduate School of Engineering, Faculty of Engineering, University of Tokyo, Hongo, Tokyo, 113-8656, Japan
[2]Ministry of Water Supply, Irrigation and Energy, Koshi Province, C7PG+924, Nepal
[3]Department of Electronics, Faculty of Engineering, Wayamba University, Kurunegala, 60170, Sri Lanka
[4]Department of Civil Engineering and Construction, Faculty of Engineering and Design, Atlantic Technological University, Sligo, F91 YW50, Ireland
[5]School of Systems Engineering, Kochi University of Technology, 185 Miyanokuchi, Tosayamada, Kami City, Kochi 782-8502, Japan

Contents

List of Tables

1 Experiment D - Model Specifications 2

Sup.Table 1: Experiment D - Model Specifications

Model Number	Model	Prediction Speed (obs/sec)	Training Time (sec)	Compact Model Size (bytes)	Coder Model Size (bytes)
1	Bagged Trees	4974.78	3.19	5580	1287
2	Bilayered Neural Network	5589.64	2.55	4129	1222
3	Boosted Trees	5499.14	3.90	2869	692
4	Coarse Gaussian SVM	5543.81	1.24	2365	480
5	Coarse Tree	5103.13	3.43	4986	2122
6	Cubic SVM	5175.42	2.64	4970	2122
7	Efficient Linear Least Squares	4968.40	2.62	4970	2122
8	Efficient Linear SVM	5094.85	2.57	4902	2058
9	Exponential GPR	5101.44	2.57	4390	1546
10	Fine Gaussian SVM	5225.99	2.50	4582	1738
11	Fine Tree	5497.81	2.48	9649	776
12	Least Squares Regression Kernel	5486.09	2.52	9689	776
13	Linear	1741.98	2.57	98910	15530
14	Linear SVM	1551.25	2.80	98682	15448
15	Matern 5/2 GPR	3760.35	2.85	9360	4220
16	Medium Gaussian SVM	5082.98	2.58	9340	4200
17	Medium Neural Network	5096.48	2.64	9346	4206
18	Medium Tree	4229.45	2.92	9391	4226
19	Narrow Neural Network	5203.17	2.93	5344	1908
20	Quadratic SVM	5090.93	2.76	5944	2508
21	Rational Quadratic GPR	4773.85	2.80	8944	5508
22	Squared Exponential GPR	5200.46	4.58	7116	3156
23	SVM Kernel	5097.99	2.79	8888	4404
24	Trilayered Neural Network	3866.25	3.27	8491	3448
25	Wide Neural Network	4591.71	2.63	8421	3448
